# List-Based Simulated Annealing Algorithm for Traveling Salesman Problem

**DOI:** 10.1155/2016/1712630

**Published:** 2016-03-13

**Authors:** Shi-hua Zhan, Juan Lin, Ze-jun Zhang, Yi-wen Zhong

**Affiliations:** ^1^College of Computer and Information Science, Fujian Agriculture and Forestry University, Fuzhou 350002, China; ^2^Center of Modern Education Technology and Information Management, Fujian Agriculture and Forestry University, Fuzhou 350002, China

## Abstract

Simulated annealing (SA) algorithm is a popular intelligent optimization algorithm which has been successfully applied in many fields. Parameters' setting is a key factor for its performance, but it is also a tedious work. To simplify parameters setting, we present a list-based simulated annealing (LBSA) algorithm to solve traveling salesman problem (TSP). LBSA algorithm uses a novel list-based cooling schedule to control the decrease of temperature. Specifically, a list of temperatures is created first, and then the maximum temperature in list is used by Metropolis acceptance criterion to decide whether to accept a candidate solution. The temperature list is adapted iteratively according to the topology of the solution space of the problem. The effectiveness and the parameter sensitivity of the list-based cooling schedule are illustrated through benchmark TSP problems. The LBSA algorithm, whose performance is robust on a wide range of parameter values, shows competitive performance compared with some other state-of-the-art algorithms.

## 1. Introduction

Simulated annealing (SA) algorithm, which was first independently presented as a search algorithm for combinatorial optimization problems in [1, 2], is a popular iterative metaheuristic algorithm widely used to address discrete and continuous optimization problems. The key feature of SA algorithm lies in means to escape from local optima by allowing hill-climbing moves to find a global optimum. One of the major shortages of SA is that it has several parameters to be tuned, and its performance is sensitive to the values of those control parameters. There are two special strategies for parameter tuning: the online parameter tuning and the off-line parameter tuning. In the online approach, the parameters are controlled and updated dynamically or adaptively throughout the execution of the SA algorithm [3–7], whereas in the off-line approach, the values of different parameters are tuned before the execution of the SA algorithm and are fixed during its execution. Fine-tuning parameter values is not trivial, and those parameters are quite often very poorly made by trial and error. So, SA algorithm, which has less parameter or is less sensitive to parameter setting, is very attractive for practical users.

Recently, a metaheuristic algorithm called the list-based threshold-accepting (LBTA) algorithm has been developed and has shown significant performance for combinatorial optimization problems that are NP-complete. The advantage of LBTA over the majority of other neighbourhood search-based metaheuristic methods is that it has fewer controlling parameters that have to be tuned in order to produce satisfactory solutions. Since its appearance, LBTA has been successfully applied to many combinatorial optimization problems [8–14].

In this work we are motivated by the success of LBTA in simplifying parameter tuning to study how list-based parameter control strategy can be applied to SA algorithm. Towards by this goal, our paper presents a novel list-based cooling schedule for SA algorithm to solve travelling salesman problem (TSP), and we call our proposed algorithm as list-based simulated annealing (LBSA) algorithm. In list-based cooling schedule, all temperatures are stored in a list which is organized as a priority queue. A higher temperature has higher priority. In LBSA, a list of temperatures is created first, and then, in each generation, the maximum value in the list is used as current temperature to calculate acceptance probability for candidate solution. The temperature list is updated adaptively according to the topology of the solution space of the problem. Using the list-based cooling schedule, SA algorithm has good performance on a wide range of parameter values; and it also has competitive performance compared with some other state-of-the-art algorithms. The parameter robustness of list-based cooling schedule can greatly simplify the design and implementation of LBSA algorithm for practical applications.

The remainder of this paper is organized as follows: Section 2 provides a short description of TSP problem and SA algorithm.  Section 3 presents our proposed list-based SA algorithm.  Section 4 gives the experimental approach and results of experiments carried out on benchmark TSP problems. Finally, in Section 5 we summarize our study.

## 2. Preliminaries

### 2.1. Traveling Salesman Problem

TSP problem is one of the most famous hard combinatorial optimization problems. It belongs to the class of NP-hard optimization problems. This means that no polynomial time algorithm is known to guarantee its global optimal solution. Consider a salesman who has to visit *n* cities. The TSP problem consists of finding the shortest tour through all the cities such that no city is visited twice and the salesman returns back to the starting city at the end of the tour. It can be defined as follows. For *n* cites problem, we can use a distance matrix *D* = (*d*_*i*,*j*_)_*n*×*n*_ to store distances between all the pair of cites, where each element *d*_*i*,*j*_ of matrix *D* represents the distance between cities *v*_*i*_ and *v*_*j*_. And we use a set of permutations *π* of the integers from 1 to *n*, which contains all the possible tours of the problem. The goal is to find a permutation *π* = (*π*(1), *π*(2),…, *π*(*n*)) that minimizes

(1) $$\begin{array}{c} f\left(\;\pi \;\right)=\overset{n-1}{\underset{i=1}{\sum }}d_{\pi \left(i\right),\pi \left(i+1\right)}+d_{\pi \left(n\right),\pi \left(1\right)}. \end{array}$$

TSP problem may be symmetric or asymmetric. In the symmetric TSP, the distance between two cities is the same in each opposite direction, forming an undirected graph. This symmetry halves the number of possible solutions. In the asymmetric TSP, paths may not exist in both directions or the distances might be different, forming a directed graph. Traffic collisions, one-way streets, and airfares for cities with different departure and arrival fees are examples of how this symmetry could break down.

### 2.2. Simulated Annealing Algorithm

**Figure 1:**
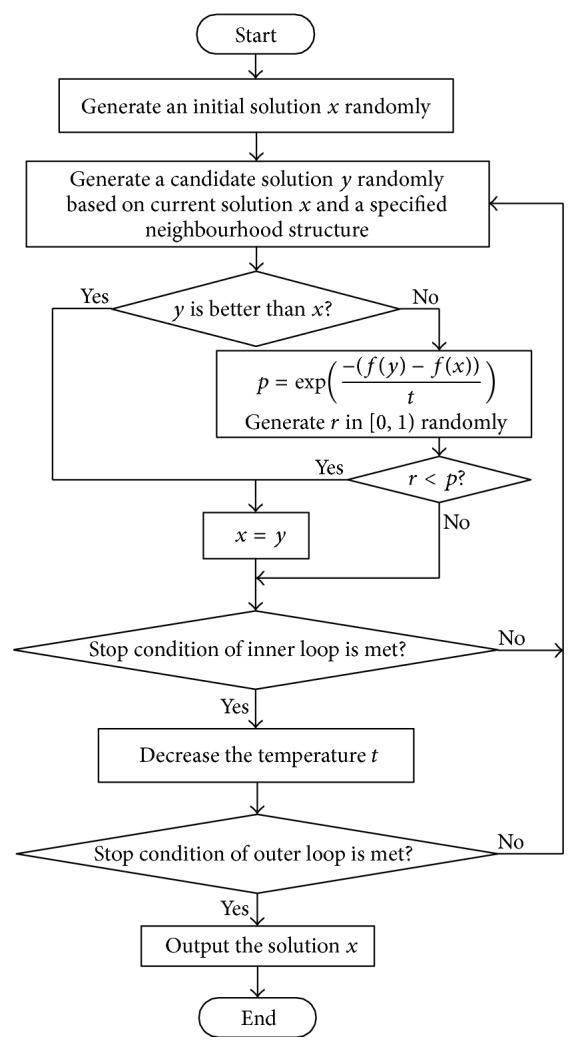
Flowchart of simulated annealing algorithm.

SA algorithm is commonly said to be the oldest among the metaheuristics and surely one of the few algorithms that have explicit strategies to avoid local minima. The origins of SA are in statistical mechanics and it was first presented for combinatorial optimization problems. The fundamental idea is to accept moves resulting in solutions of worse quality than the current solution in order to escape from local minima. The probability of accepting such a move is decreased during the search through parameter temperature. SA algorithm starts with an initial solution *x*, and candidate solution *y* is then generated (either randomly or using some prespecified rule) from the neighbourhood of *x*. The Metropolis acceptance criterion [15], which models how a thermodynamic system moves from one state to another state in which the energy is being minimized, is used to decide whether to accept *y* or not. The candidate solution *y* is accepted as the current solution *x* based on the acceptance probability:

(2) $$\begin{array}{c} p=\left\{\begin{array}{cc} 1, & \text{if }f\left(y\right)\leq f\left(x\right),\\ e^{-\left(f\left(y\right)-f\left(x\right)\right)/t}, & \text{otherwise}, \end{array}\right. \end{array}$$

where *t* is the parameter temperature. The SA algorithm can be described by Figure 1.

In order to apply the SA algorithm to a specific problem, one must specify the neighbourhood structure and cooling schedule. These choices and their corresponding parameter setting can have a significant impact on the SA's performance. Unfortunately, there are no choices of these strategies that will be good for all problems, and there is no general easy way to find the optimal parameter sets for a given problem. A cooling schedule should consist of starting temperature, temperature decrement function, Markov chain length, and termination condition. Geometric cooling schedule, which can be described by the temperature-update formula *t*_*k*+1_ = *at*_*k*_, is probably the most commonly used in the SA literature and acts as a base line for comparison with other more elaborate schemes [16]. Typical values of *a* for moderately slow cooling rates are 0.8 through 0.99.

For practical application where computation complexity of objective function is high, SA algorithm may run with constant Markov chain length and use fixed iteration times as termination condition. As a result, initial temperature and cooling coefficient *a* are the two key parameters that impact the performance of SA algorithm. Even in this simple situation, it is not an easy task to find optimal values for those two parameters, because SA's performance is sensitive to those parameters. One way to tackle the parameter setting problem of SA algorithm is to use adaptive cooling strategy [3–7]. Adaptive cooling strategy is definitely efficient and it makes SA algorithm less sensitive to user defined parameters than canonical SA, but it also makes SA algorithm lose the feature of simplicity. Another way to tackle this problem is to find new cooling schedule which has fewer parameters or the parameters are more robust.

### 2.3. Simulated Annealing Algorithm for TSP Problems

In order to use SA algorithm for TSP problem, we can represent solution *x* as a permutation *π*. Then, a large set of operators, such as the famous 2-Opt, 3-Opt, inverse, insert, and swap operators, can be used to generate candidate solution *y*. Since its appearance, SA algorithm has been widely and deeply studied on TSP problems [17–21]. Many cooling schedules, such as geometric, exponential, logarithmic, and arithmetic ones and their variants, have been used in literature.

The theory convergence conditions of SA make it very time consuming in most cases [22]. Wang et al. [23] proposed a two-stage SA algorithm which was tested on 23 TSP benchmark instances with scale from 51 to 783. The numerical results show that it is difficult for SA algorithm to solve TSP benchmark instances with scale exceeding 1000 cities based on time complexity. Geng et al. [24] proposed an adaptive simulated annealing algorithm with greedy search (ASA-GS), where greedy search technique is used to speed up the convergence rate. The ASA-GS achieves a reasonable trade-off among computation time, solution quality, and complexity of implementation. It has good scalability and has good performance even for TSP benchmark instances with scale exceeding 1000 cities. Wang et al. [25] proposed a multiagent SA algorithm with instance-based sampling (MSA-IBS) by exploiting learning ability of instance-based search algorithm. The learning-based sampling can effectively improve the SA's sampling efficiency. Simulation results showed that the performance of MSA-IBS is far better than ASA-GS in terms of solution accuracy and CPU time. In this paper, MSA-IBS is used as basis to use list-based cooling schedule.

## 3. List-Based Simulated Annealing Algorithm

### 3.1. The Neighbourhood Structure

In this paper, we use the greedy hybrid operator proposed by Wang et al. [25] to produce candidate solution. This is a kind of multiple move operators, which select the best one from three neighbours. Specifically, after two positions *i* and *j* are selected, it uses inverse operator, insert operator, and swap operator to produce three neighbour solutions. And the best one is used as the candidate solution. The inverse, insert, and swap operators can be defined as follows.

**Define 1 (inverse operator inverse(*π*, *i*, *j*)).**

It means to inverse the cities between positions *i* and *j*. The inverse(*π*, *i*, *j*) will generate a new solution *π*′ such that *π*′(*i*) = *π*(*j*), *π*′(*i* + 1) = *π*(*j* − 1),…, *π*′(*j*) = *π*(*i*), where 1 ≤ *i*, *j* ≤ *n*∧1 ≤ *j* − *i* < *n* − 1; in addition, if *j* − *i* = *n* − 1, it means *i* = 1 and *j* = *n*, and then *π*′(*i*) = *π*(*j*) and *π*′(*j*) = *π*(*i*). Two edges will be replaced by inverse operator for symmetric TSP problems.

**Define 2 (insert operator insert(*π*, *i*, *j*)).**

It means to move the city in position *j* to position *i*. The insert(*π*, *i*, *j*) operator will generate a new solution *π*′ such that *π*′(*i*) = *π*(*j*), *π*′(*i* + 1) = *π*(*i*),…, *π*′(*j*) = *π*(*j* − 1), in the case of *i* < *j*, or *π*′(*j*) = *π*′(*j* + 1),…,  *π*′(*i* − 1) = *π*(*i*),  *π*′(*i*) = *π*(*j*), in the case of *i* > *j*. In general, three edges will be replaced by insert operator.

**Define 3 (swap operator swap(*π*, *i*, *j*)).**

It means to swap the city in position *j* and city in position *i*. The swap(*π*, *i*, *j*) operator will generate a new solution *π*′ such that *π*′(*i*) = *π*(*j*) and *π*′(*j*) = *π*(*i*). In general, four edges will be replaced by swap operator.

Using the above three operators, the used hybrid greedy operator can be defined as follows:

(3) π′=min⁡inverseπ,i+1,j,insertπ,i+1,j,swapπ,i+1,j,

where min returns the best one among its parameters.

### 3.2. Produce Initial Temperature List

As in LBTA algorithms [8–14], list-based parameter controlling strategy needs to produce an initial list of parameters. Because temperature in SA is used to calculate acceptance probability of candidate solution, we use initial acceptance probability *p*_0_ to produce temperature *t* as follows. Suppose *x* is current solution and *y* is candidate solution which is randomly selected from *x*'s neighbours. Their objective function values are *f*(*x*) and *f*(*y*), respectively. If *y* is worse than *x*, then the acceptance probability *p* of *y* can be calculated using formula (2). Conversely, if we know the acceptance probability *p*_0_, then we can calculate the corresponding temperature *t* as follows:

(4) $$\begin{array}{c} t=\frac{-\left(f\left(y\right)-f\left(x\right)\right)}{\ln\left(p_{0}\right)}. \end{array}$$

**Figure 2:**
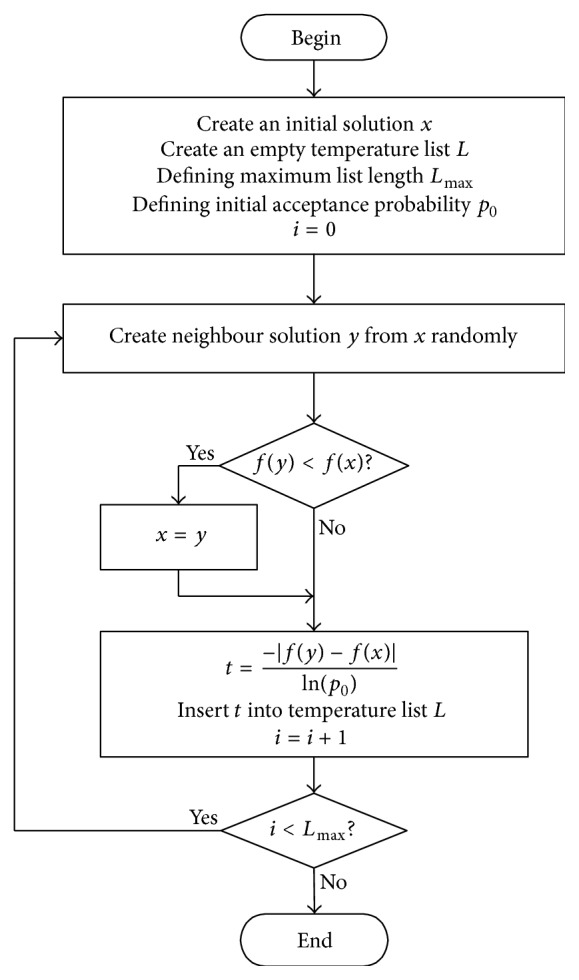
Flowchart of producing initial temperature list.

Figure 2 is the flowchart of producing initial temperature list. In Figure 2, after a feasible solution *x* is produced, a candidate solution *y* is randomly selected from *x*'s neighbours. If *y* is better than *x*, then *x* is replaced by *y*. And using formula (4), the absolute value of *f*(*y*) − *f*(*x*) is used to calculate an initial temperature value *t*. Then *t* will be inserted into initial temperature list. The temperature list is a priority list, where higher temperature has higher priority. The same procedure is repeated until the list of temperature values is filled.

### 3.3. Temperature Controlling Procedure

In each iteration *k*, the maximum temperature in list is used as current temperature *t*_max_. If Markov chain length is *M*, then *t*_max_ may be used at best *M* times for the calculation of acceptance probability of candidate solution. Suppose there are *n* times that bad solution is accepted as current solution; we use *d*_*i*_ and *p*_*i*_ to represent the difference of objective function values and acceptance probability, respectively, where *i* = 1 ⋯ *n*, and the relation between *d*_*i*_ and *p*_*i*_ can be described as the following equation:

(5) $$\begin{array}{c} p_{i}=e^{-d_{i}/t_{max}}. \end{array}$$

According to the Metropolis acceptance criterion, for each time a bad candidate solution is met, a random number *r* is created. If *r* is less than the acceptance probability, then the bad candidate solution will be accepted. So, for each pair of *d*_*i*_ and *p*_*i*_, there is a random number *r*_*i*_ and *r*_*i*_ is less than *p*_*i*_. We use *d*_*i*_ and *r*_*i*_ to calculate a new temperature *t*_*i*_ as the following formula described:

(6) $$\begin{array}{c} t_{i}=\frac{-d_{i}}{\ln\left(r_{i}\right)}. \end{array}$$

We use the average of *t*_*i*_ to update the temperature list. Specifically, *t*_max_ will be removed from list, and average of *t*_*i*_ will be inserted into list. Because *t*_*i*_ is always less than *t*_max_, the average of *t*_*i*_ is less than *t*_max_ also. In this way, the temperature decreases always as the search proceeds.

### 3.4. Description of LBSA Algorithm

**Figure 3:**
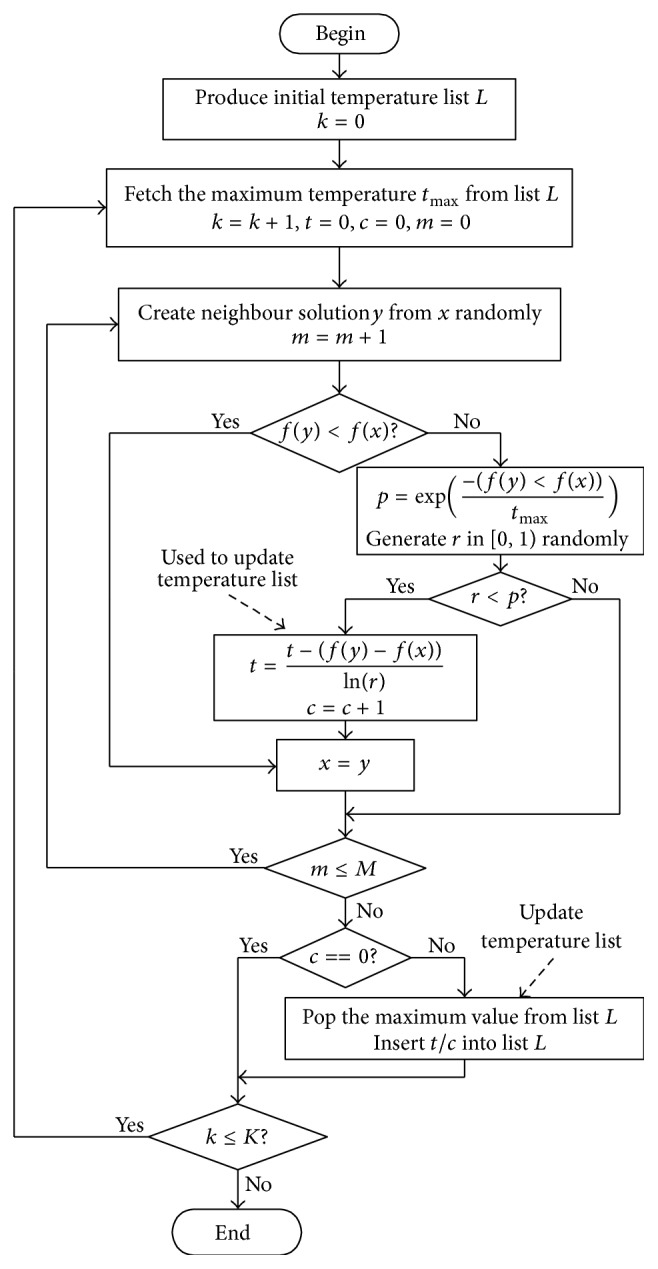
Flowchart of list-based SA algorithm.

Because the main purpose of this paper is to study the effectiveness of list-based cooling schedule, we use a simple SA framework which uses fixed iteration times for outer loop and fixed Markov chain length in each temperature. The detailed flowchart of the main optimization procedure is shown in Figure 3. Besides the creation of initial temperature list, the main difference between the flowchart of LBSA and the flowchart of canonical SA is about the temperature control strategy. In canonical SA, the temperature controlling procedure is independent of the topology of solution space of the problem. Conversely, the temperature controlling procedure of LBSA is adaptive according to the topology of solution space of the problem. In Figure 3, *K* is maximum iteration times of outer loop, which is termination condition of LBSA. *M* is Markov chain length, which is termination condition of inner loop. Counter *k* is used to record the current iteration times of outer loop, *m* is used to record current sampling times of inner loop, and *c* is used to record how many times bad solution is accepted in each temperature. Variable *t* is used to store the total temperature calculated by formula (6), and the average temperature *t*/*c* will be used to update the temperature list. We can use a maximum heap to implement the temperature list. As the time complexity of storage and retrieval from heap is logarithmic, the use of temperature list will not increase the time complexity of SA algorithm.

## 4. Simulation Results

In order to observe and analyse the effect of list-based cooling schedule and the performance of LBSA algorithm, five kinds of experiments were carried out on benchmark TSP problems. The first kind of experiments was used to analyse the effectiveness of the list-based cooling schedule. The second kind was carried out to analyse the parameter sensitivity of LBSA algorithm. The third kind was carried out to compare the performance of different ways to update the temperature list. The fourth kind was carried out to compare the performance of different neighbourhood structures. And the fifth kind was carried out to compare LBSA's performance with some of the state-of-the-art algorithms.

### 4.1. The Effectiveness of List-Based Cooling Schedule

To illustrate the effectiveness of list-based cooling schedule, we compare the temperature varying process and search process of LBSA algorithm with SA algorithms based on the classical geometric cooling schedule and arithmetic cooling schedule. Those experiments were carried out on BCL380, XQL662, XIT1083, and XSC6880 problems from VLSI data sets. The best known integer solutions of those problems are 1621, 2513, 3558, and 21537, respectively. The iteration times of outer loop are 1000, and the Markov chain length in each temperature is the city number of the problem, which is 380, 662, 1083, and 6880, respectively.

**Figure 4:**
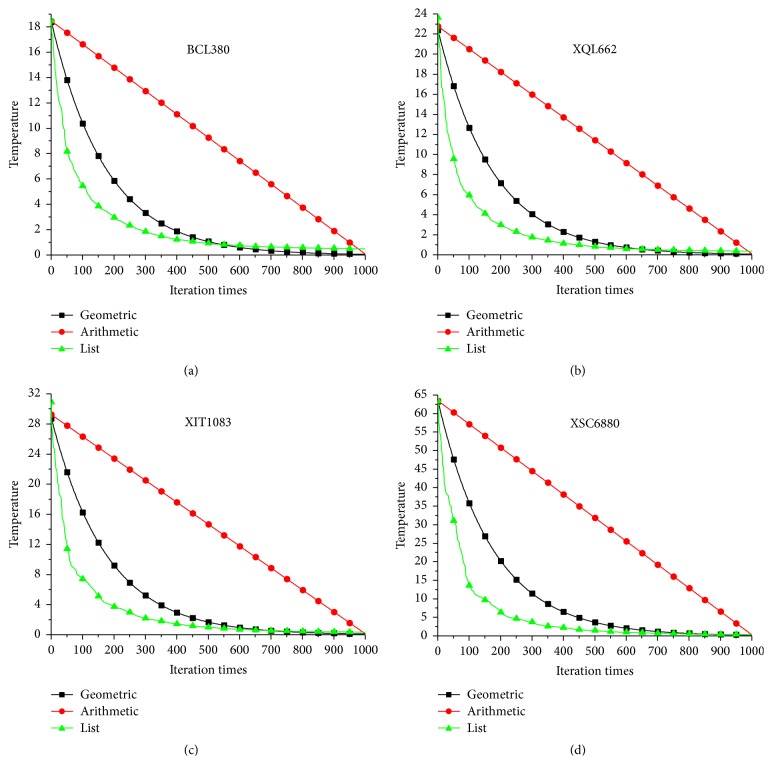
Compare the temperature varying process of different cooling schedule.

Figure 4 compares the temperature varying process of different cooling schedules on the four benchmark problems. List-based cooling schedule can be viewed as a kind of geometric cooling schedule with variable cooling coefficient. Compared with the temperature varying of geometric cooling schedule, temperature of list-based cooling schedule decreases quicker in the early stage, but slower in the later stage. As indicated by Abramson et al. [16], geometric cooling schedule always uses constant cooling coefficient regardless of the stages of search. However, at high temperatures almost all candidate solutions are accepted, even though many of them could be nonproductive. To use variable cooling coefficients, which depend on the stages, would allow SA algorithm to spend less time in the high temperature stages. Consequently, more time would be spent in the low temperature stages, thus reducing the total amount of time required to solve the problem.

**Figure 5:**
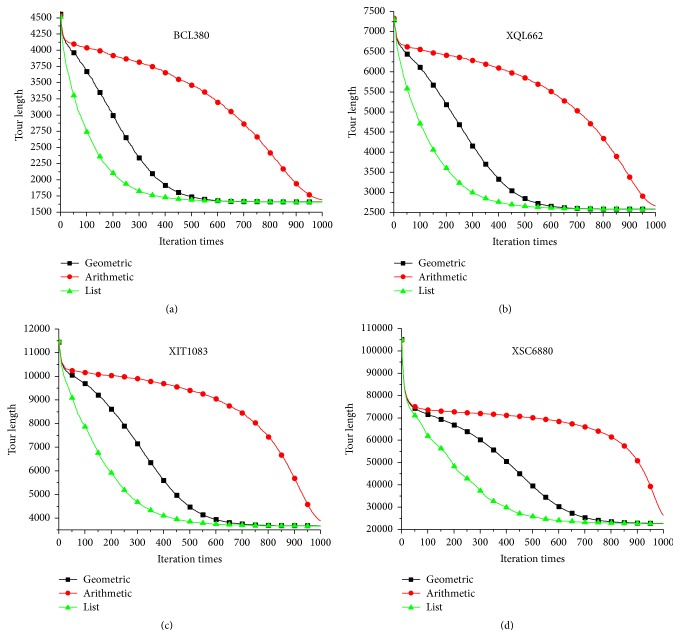
Compare the search process of different cooling schedule.

Figure 5 compares the search process of different cooling schedules on the four benchmark problems. Compared with geometric cooling schedule and arithmetic cooling schedule, list-based cooling schedule has quicker convergence speed and has good long-term behaviour also. This good performance may be due to the variable cooling coefficient feature of list-based cooling schedule. The temperature is updated adaptively according to the topology of solution space of the problem. As a result, LBSA algorithm can spend more time to search on promising area finely.

### 4.2. Sensitivity Analysis of Temperature List Length and Initial Temperature Values

**Figure 6:**
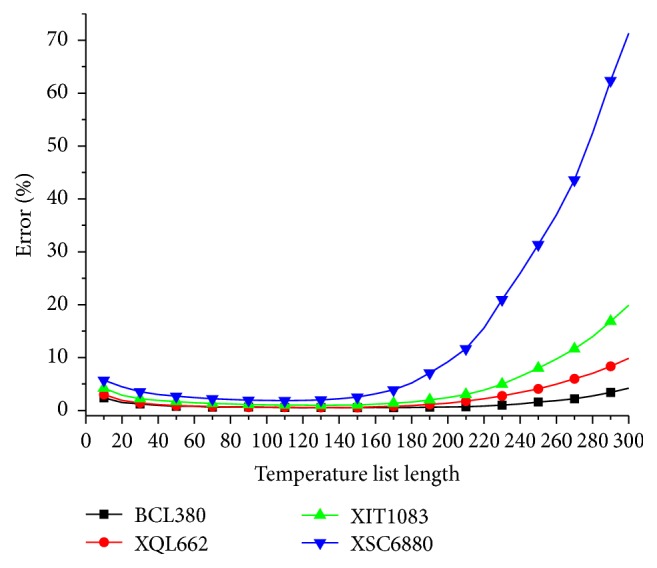
Sensitivity of parameter temperature list length.

We observe the sensitivity of list length on BCL380, XQL662, XIT1083, and XSC6880 problems. For each problem, we test 30 different list lengths from 10 to 300 with a step 10. For each list length, we run LBSA algorithm 50 times and calculate the average tour length and the percentage error of the mean tour length to the best known tour length.  Figure 6 is the relation between percentage error and list length. It shows the following: (1) for all the problems, there is a wide range of list length for LBSA to have similar promising performance; (2) list length is more robust on small problems than on large problems; (3) list length should not be too big for large problems. For big problems, the used Markov chain length, which is the city number of the problem, is not big enough for LBSA to reach equilibrium on each temperature. Big list length means the temperature decreases more slowly, so the algorithm will spend too much time on high temperature and accept too much nonproductive solutions. As a result, a big list length will dramatically deteriorate LBSA's performance for large problems. Because of the robust temperature list length, we use fixed temperature list length in the following simulations, which is 120 for all instances.

**Figure 7:**
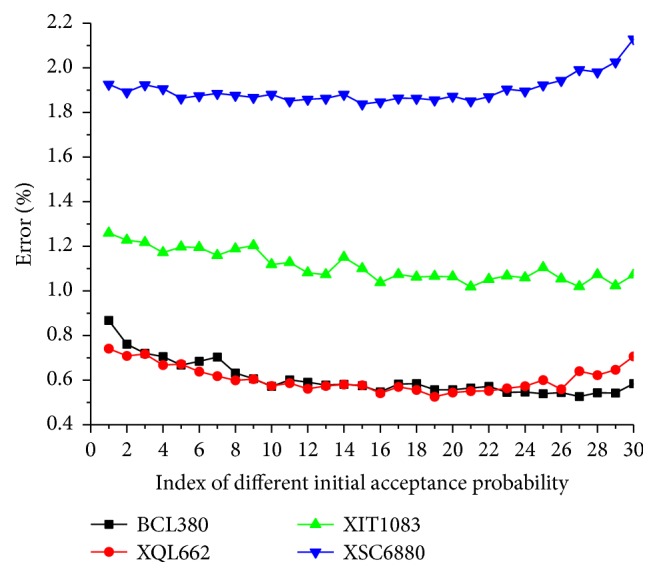
Sensitivity of parameter initial temperature values.

In order to observe the sensitivity of initial temperature values, we carried out experiments with different initial temperature values on BCL380, XQL662, XIT1083, and XSC6880 problems. Because the initial temperature values are produced according to initial acceptance probability *p*_0_, we use 30 different *p*_0_, which range from 10^−20^ to 0.9, to create initial temperature values. Specifically, the set of *p*_0_ is the union of geometric sequence from 10^−20^ to 10^−2^ with common ratio 10 and arithmetic sequence from 0.1 to 0.9 with common difference 0.1. For each initial acceptance probability, we run LBSA algorithm 50 times and calculate the average tour length and the percentage error of the mean tour length to the best known tour length.  Figure 7 is the relation between percentage error and index of initial acceptance probability. It shows the following: (1) the performance of LBSA is not sensitive to the initial temperature values; (2) there is a different varying direction among different problems. For small problems, the initial temperature should not be too low, but for big problems, the initial temperature should not be too high. This difference is due to the limited computation resource used and the nonlinear computation complexity of TSP problem. The LBSA algorithm can have similar promising performance on a wide range of initial temperature values; this high robustness of initial temperature is due to the adaptive nature of list-based cooling schedule.

### 4.3. Comparing Different Temperature Updating Schemes

**Figure 8:**
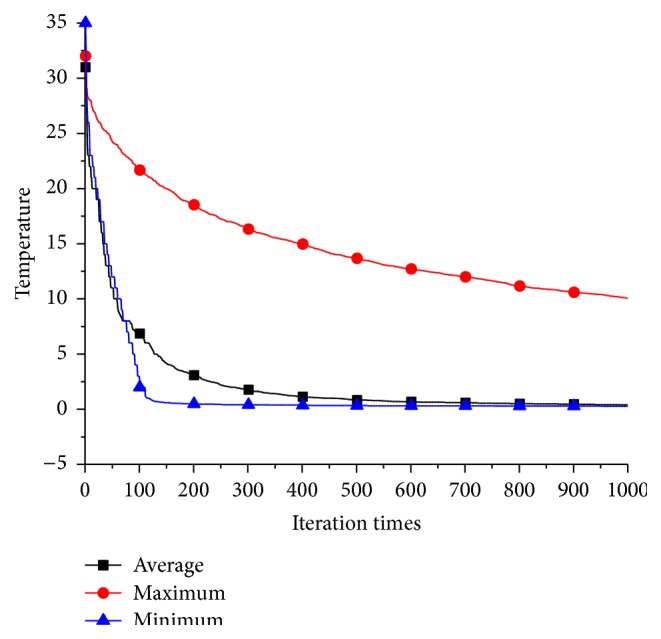
Temperature decreasing process of different temperature list updating schemes on instance BCL380.

In our algorithm described in Section 3.4, we use the average of temperature *t*_*i*_ to update temperature list. There are several variants, such as using maximum *t*_*i*_ or minimum *t*_*i*_. To show the advantage of using average of *t*_*i*_, we compare the results and the decreasing process of temperature using those three updating schemes.  Table 1 is the simulation results; it is clear that using average temperature to update temperature list has far better performance than the other methods. The temperature decreasing process of different strategies on BCL380, which is showed in Figure 8, can explain why using average temperature is the best option. If we use maximum temperature to update the temperature list, the temperature will decrease very slowly. As a result, the acceptation probability of bad solution is always high, and SA algorithm will search randomly in the solution space. Conversely, if we use minimum temperature to update the temperature list, the temperature will decrease sharply. As a result, the SA algorithm will be trapped into local minimum quickly and lose the advantage of escaping from local minimum by using Metropolis acceptance criterion.

### 4.4. Comparing Different Neighbourhood Structures

**Figure 9:**
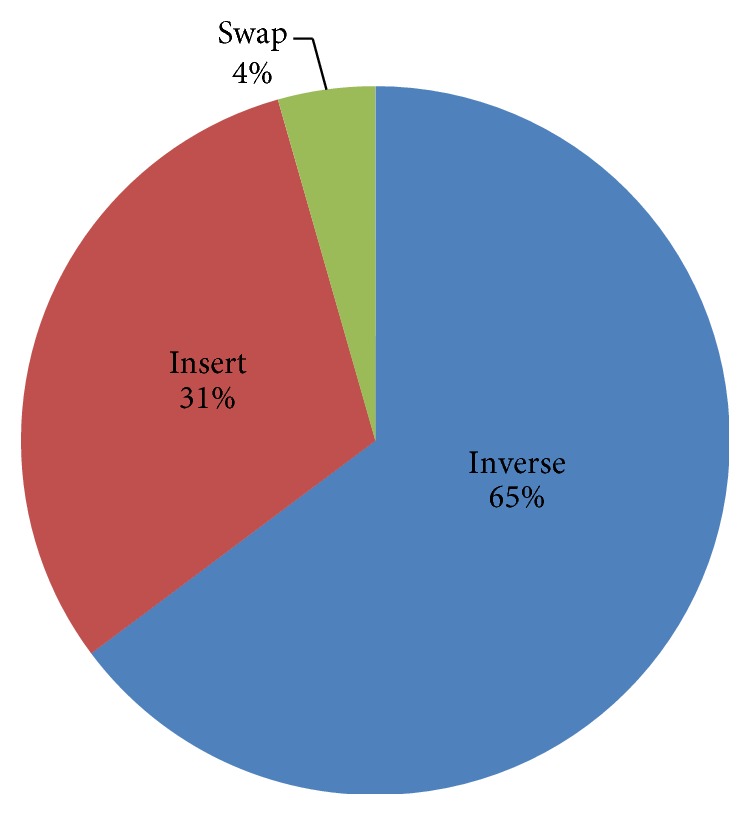
Percentage of inverse, insert, and swap operators accepted in hybrid operator on instance BCL380.

In our algorithm, we use a greedy hybrid neighbour operator proposed by Wang et al. [25] to produce candidate solution. This hybrid operator uses the best one produced by inverse, insert, and swap operators. To show its advantage, we compare the results produced by inverse, insert, swap, and hybrid operators. And we compare the percentage of inverse, insert, and swap operators accepted when we use hybrid operator.  Table 2 is the simulation results; it is clear that hybrid operator has the best performance. Among the three basic operators, inverse is the best. If we compare the percentages of different basic operators accepted when we use hybrid operator, we found that inverse operator is accepted most.  Figure 9 is the percentages of different operators accepted on instance BCL380. The percentages of inverse, insert, and swap operators are 65%, 31%, and 4%, respectively. The relative percentages of the three operators accepted are similar on other instances as on BCL380. The good performance of inverse operator is due to its ability to produce more fine-grained neighbourhood structure, because it changes two edges only. The hybrid operator, which uses inverse, insert, and swap operators at the same time, has a bigger neighbourhood structure. So it has higher probability to find promising solutions.

### 4.5. Competitiveness of LBSA Algorithm

We compare LBSA algorithm with genetic simulated annealing ant colony system (GSAACS) [26] and MSA-IBS on 24 benchmark instances with cities from 51 to 1655. The GSAACS is a hybrid algorithm which uses the ant colony system (ACS) to generate the initial solutions of the genetic algorithms. Then, it uses genetic algorithm (GA), which uses SA as mutation operator, to generate offspring solutions based on the initial solutions. If the solutions searched by GA are better than the initial solutions, GSAACS will use these better solutions to update the pheromone for the ACS. After a predefined number of generations, GSAACS uses particle swarm optimization (PSO) to exchange the pheromone information between groups.

The results of GSAACS are from [26]. GSAACS uses 120 ants and 1000 generations. In each generation of GSAACS, GA runs 100 generations. In MSA-IBS and LBSA algorithm, we set the population size to 30 and the iteration times of outer loop to 1000, and the Markov chain length in each temperature is two times the number of cities. The end condition is that either it finds the optimal solution or the iteration times of outer loop reach 1000. The algorithm was executed 25 times on each TSP problem, and the results are listed in Table 3.

In Table 3, the columns Instance, OPT, Best, Mean, and PEav denote the name of the TSP problem, the optimal tour length from TSPLIB, the shortest tour length found, the average tour length among the 25 trials, and the percentage error of the mean tour length to the OPT, respectively. As can be seen in Table 3, both MSA-IBS and LBSA have better performance than GSAACS on all 24 instances. LBSA is a little better than MSA-IBS in terms of average percentage error.

We compare LBSA algorithm with GA-PSO-ACO [27] and MSA-IBS on 35 benchmark instances with cities from 48 to 33810. GA-PSO-ACO combines the evolution ideas of the genetic algorithms, particle swarm optimization, and ant colony optimization. In the GA-PSO-ACO algorithm, the whole process is divided into two stages. In the first stage, GA and PSO are used to obtain a series of suboptimal solutions to adjust the initial allocation of pheromone for ACO. In the second stage, ACO is used to search the optimal solution. The results of GA-PSO-ACO are from [27]. GA-PSO-ACO uses 100 individuals and 1000 generations. In LBSA and MSA-IBS algorithm, we set the number of population size to 10 and the iteration times of outer loop to 1000, and the Markov chain length in each temperature is the number of cities. The end condition of LBSA is that either it finds the optimal solution or the iteration times of outer loop reach 1000. Like GA-PSO-ACO and MSA-IBS, LBSA was executed 20 times on each TSP instance, and the results are listed in Table 4.

As can be seen in Table 4, both MSA-IBS and LBSA have better performance than GA-PSO-ACO on all 35 instances. LBSA is a little bit better than MSA-IBS in terms of average percentage error.

We compare LBSA algorithm with ASA-GS [24] and MSA-IBS algorithm on 40 benchmark instances with cities from 151 to 85900. The experiments were run on a 2.8 GHz PC, and the ASA-GS was run on a 2.83 GHz PC. For all instances, we set the iteration times of outer loop to 1000 and set the Markov chain length in each temperature to the number of cities. As in MSA-IBS algorithm, a suitable population size is selected for each instance such that the CPU time of LBSA is less than that of ASA-GS. The end condition of LBSA and MSA-IBS is either when it finds the optimal solution or when the iteration times of outer loop reach 1000. The algorithm is executed 25 trials on each instance, and the results are listed in Table 5.

As can be seen in Table 5, the average PEav of LBSA for all instances is 0.75, which is better than 1.87 of ASA-GS, and the average CPU time of LBSA for all instances is 282.49, which is far less than 650.31 of ASA-GS. LBSA is a little bit better than MSA-IBS in terms of average PEav.

## 5. Conclusion

This paper presents a list-based SA algorithm for TSP problems. The LBSA algorithm uses novel list-based cooling schedule to control the decrease of temperature parameter. The list-based cooling schedule can be viewed as a special geometric cooling schedule with variable cooling coefficient. The variance of cooling coefficient is adaptive according to the topology of solution space, which may be more robust to problems at hand. Simulated results show that this novel cooling schedule is insensitive to the parameter values and the proposed LBSA algorithm is very effective, simple, and easy to implement. The advantage of insensitive parameter is very attractive, allowing LBSA algorithm to be applied in diverse problems without much effort on tuning parameters to produce promising results.

## Figures and Tables

**Table 1:** Comparison of different temperature list updating schemes.

Instance	OPT	Average	Maximum	Minimum
BCL380	1621	**1630.2**	2357.4	1651.9
XQL662	2513	**2527.2**	4117.8	2565.8
XIT1083	3558	**3592.9**	6663.0	3674.6
XSC6880	21537	**21920.0**	63472.2	22649.8

**Table 2:** Comparison of different neighbourhood structures.

Instance	Inverse	Insert	Swap	Hybrid
BCL380	1631.96	1776.24	2666.44	**1630.84**
XQL662	2535.2	2836.52	4437.2	**2527.2**
XIT1083	3603.48	4118.72	6760.84	**3592.08**
XSC6880	22115.6	29048	64263.4	**21930.6**

**Table 3:** Compare LBSA with GSAACS and MSA-IBS on 24 benchmark instances from TSPLIB.

Number	Instance	OPT	GSAACS			MSA-IBS			LBSA		
			Best	Mean	PEav	Best	Mean	PEav	Best	Mean	PEav
1	eil51	426	427	427.27	0.30	426	426	0	426	426	0
2	eil76	538	538	540.20	0.41	538	538	0	538	538	0
3	eil101	629	630	635.23	0.99	629	629	0	629	629	0
4	berlin52	7542	7542	7542.00	0	7542	7542	0	7542	7542	0
5	bier127	118282	118282	119421.83	0.96	118282	118291.2	0.01	118282	118282	0
6	ch130	6110	6141	6205.63	1.57	6110	6110	0	6110	6110.6	0.01
7	ch150	6528	6528	6563.70	0.55	6528	6544.4	0.25	6528	6537.5	0.15
8	rd100	7910	7910	7987.57	0.98	7910	7910	0	7910	7910.1	0
9	lin105	14379	14379	14406.37	0.19	14379	14379	0	14379	14379	0
10	lin318	42029	42487	43002.90	2.32	42040	42170.9	0.34	42029	42138.55	0.26
11	kroA100	21282	21282	21370.47	0.42	21282	21282	0	21282	21284.15	0.01
12	kroA150	26524	26524	26899.20	1.41	26524	26524.15	0	26524	26524.05	0
13	kroA200	29368	29383	29738.73	1.26	29368	29383.45	0.05	29368	29371.9	0.01
14	kroB100	22141	22141	22282.87	0.64	22141	22174.2	0.15	22141	22184.2	0.2
15	kroB150	26130	26130	26448.33	1.22	26130	26134.05	0.02	26130	26137.9	0.03
16	kroB200	29437	29541	30035.23	2.03	29438	29439.4	0.01	29437	29438.65	0.01
17	kroC100	20749	20749	20878.97	0.63	20749	20749	0	20749	20749	0
18	kroD100	21294	21309	21620.47	1.53	21294	21342.75	0.23	21294	21294.55	0
19	kroE100	22068	22068	22183.47	0.52	22068	22114.4	0.21	22068	22092.6	0.11
20	rat575	6773	6891	6933.87	2.38	6813	6824.65	0.76	6789	6815.55	0.63
21	rat783	8806	8988	9079.23	3.10	8845	8869.7	0.72	8846	8866.6	0.69
22	rl1323	270199	277642	280181.47	3.69	270893	271972.3	0.66	270475	271415	0.45
23	fl1400	20127	20593	21349.63	6.07	20299	20392.4	1.32	20140	20182.2	0.27
24	d1655	62128	64151	65621.13	5.62	62786	62966.3	1.35	62454	62610.1	0.78
Average					1.62			0.25			0.15

**Table 4:** Compare LBSA with GA-PSO-ACO and MSA-IBS on 35 benchmark instances from TSPLIB.

Instance	OPT	GA-PSO-ACO			MSA-IBS			LBSA		
		Best	Mean	PEav	Best	Mean	PE	Best	Mean	PEav
Att48	33522	33524	33662	0.42	33522	33554.64	0.10	33522	33536.6	0.04
Eil51	426	426	431.84	1.37	426	426.48	0.11	426	426.5	0.12
Berlin52	7542	7544.37	7544.37	0.03	7542	7542	0	7542	7542	0
St70	675	679.60	694.60	2.90	675	677.16	0.32	675	675.55	0.08
Eil76	538	545.39	550.16	2.26	538	538.2	0.04	538	538	0
Pr76	108159	109206	110023	1.72	108159	108288	0.12	108159	108268.3	0.10
Rat99	1211	1218	1275	5.28	1211	1211.04	0.00	1211	1211.1	0.01
Rad100	7910	7936	8039	1.63	7910	7914.56	0.06	7910	7914.7	0.06
KroD100	21294	21394	21484	0.89	21294	21340.64	0.22	21294	21314.2	0.09
Eil101	629	633.07	637.93	1.42	629	629.6	0.10	629	629	0
Lin105	14379	14397	14521	0.99	14379	14380.48	0.01	14379	14379	0
Pr107	44303	44316	44589	0.65	44303	44379.88	0.17	44303	44392.25	0.20
Pr124	59030	59051	60157	1.91	59030	59032.88	0.00	59030	59031.8	0.00
Bier127	118282	118476	120301	1.71	118282	118334.6	0.04	118282	118351.2	0.06
Ch130	6110	6121.15	6203.47	1.53	6110	6121.96	0.20	6110	6127.95	0.29
Pr144	58537	58595	58662	0.21	58537	58549.72	0.02	58537	58570.15	0.06
KroA150	26524	26676	26803	1.05	26524	26538.2	0.05	26524	26542.6	0.07
Pr152	73682	73861	73989	0.42	73682	73727.96	0.06	73682	73688.8	0.01
U159	42080	42395	42506	1.01	42080	42182.32	0.24	42080	42198.85	0.28
Rat195	2323	2341	2362	1.68	2328	2334.2	0.48	2328	2331	0.34
RroA200	29368	29731	31015	5.61	29368	29422.64	0.19	29368	29405.35	0.13
Gil262	2378	2399	2439	2.57	2379	2383.56	0.23	2379	2382.45	0.19
Pr299	48191	48662	48763	1.19	48192	48263.08	0.15	48191	48250	0.12
Lin318	42029	42633	42771	1.77	42076	42292.04	0.63	42070	42264.35	0.56
Rd400	15281	15464	15503	1.45	15324	15377.56	0.63	15311	15373.75	0.61
Pcb442	50778	51414	51494	1.41	50879	51050.12	0.54	50832	51041.7	0.52
Rat575	6773	6912	6952	2.64	6819	6854.64	1.21	6829	6847.95	1.11
U724	41910	42657	42713	1.92	42150	42302.12	0.94	42205	42357.8	1.07
Rat783	8806	9030	9126	3.63	8897	8918.28	1.28	8887	8913.25	1.22
Pr1002	259045	265987	266774	2.98	261463	262211.7	1.22	261490	262202.5	1.22
D1291	50801	52378	52443	3.23	51098	51340.84	1.06	51032	51358.7	1.10
D1655	62128	64401	65241	5.01	62784	63011.96	1.42	62779	62994.65	1.39
Nl4461	182566	18933	192574	5.48	185377	185631.1	1.68	185290	185501.7	1.61
Brd14051	469385	490432	503560	7.28	478040	478618.8	1.97	477226	477612.7	1.75
Pla33810	66048945	70299195	72420147	9.65	67868250	68038833.1	3.01	67754877	67848535.1	2.72
Average				2.43			0.53			0.49

**Table 5:** Compare LBSA with ASA-GS and MSA-IBS on 40 benchmark instances from TSPLIB.

Number	Instance	OPT	ASA-GS			MSA-IBS			LBSA		
			Mean	PEav	Time	Mean	PEav	Time	Mean	PEav	Time
1	Ch150	6528	6539.8	0.16	10.91	6529	0.02	0.86	6529.8	0.03	1.29
2	Kroa150	26524	26538.6	0.05	10.9	26524	0	0.82	26524	0	0.98
3	Krob150	26130	26178.1	0.18	10.9	26135	0.02	1.51	26137	0.03	1.65
4	Pr152	73682	73694.7	0.01	10.85	73682	0	0.84	73682	0	0.87
5	U159	42080	42398.9	0.75	11.49	42080	0	0.79	42080	0	0.91
6	Rat195	2323	2348.05	1.07	14.37	2330.2	0.31	1.86	2328	0.22	1.93
7	D198	15780	15845.4	0.41	14.6	15780	0	1.39	15780	0	1.53
8	Kroa200	29368	29438.4	0.23	14.26	29378	0.03	1.74	29373.8	0.02	1.67
9	Krob200	29437	29513.1	0.25	14.24	29439.8	0.01	1.95	29442.2	0.02	2.1
10	Ts225	126643	126646	0.00	16.05	126643	0	1.3	126643	0	1.54
11	Pr226	80369	80687.4	0.39	16.7	80369	0	1.93	80369.8	0.00	2.16
12	Gil262	2378	2398.61	0.86	19.43	2378.8	0.03	2.39	2379.2	0.05	2.72
13	Pr264	49135	49138.9	0.00	19.09	49135	0	1.43	49135	0	1.49
14	Pr299	48191	48326.4	0.28	21.94	48226.4	0.07	2.67	48221.2	0.06	2.93
15	Lin318	42029	42383.7	0.84	23.35	42184.4	0.37	2.4	42195.6	0.40	2.58
16	Rd400	15281	15429.8	0.97	30.4	15347.2	0.43	3.2	15350.4	0.45	3.46
17	Fl417	11861	12043.8	1.54	32.02	11875.6	0.12	3.72	11867.8	0.06	4.01
18	Pr439	107217	110226	2.80	34.92	107407.2	0.18	3.6	107465.2	0.23	3.95
19	Pcb442	50778	51269.2	0.96	35.75	50970	0.38	3.68	50877	0.19	4.31
20	U574	36905	37369.8	1.25	48.47	37155.8	0.68	5.21	37164.6	0.70	6.13
21	Rat575	6773	6904.82	1.94	52.1	6839.8	0.99	5.27	6837.4	0.95	5.99
22	U724	41910	42470.4	1.33	66.83	42212.2	0.72	8.11	42252	0.82	8.34
23	Rat783	8806	8982.19	2.00	78.9	8893.4	0.99	8.99	8888.2	0.93	8.9
24	Pr1002	259045	264274	2.01	164.42	261481.8	0.94	12.71	261805.2	1.07	12.96
25	Pcb1173	56892	57820.5	1.63	193.08	57561.6	1.18	8.9	57431.8	0.95	9.61
26	D1291	50801	52252.3	2.85	214.64	51343.8	1.07	10.59	51198.8	0.78	11.77
27	Rl1323	270199	273444	1.20	210.16	271818.4	0.6	11.53	271714.4	0.56	12.64
28	Fl1400	20127	20782.2	3.25	232.02	20374.8	1.23	17.72	20249.4	0.61	15.43
29	D1655	62128	64155.9	3.26	281.88	62893	1.23	16.18	63001.4	1.41	16.45
30	Vm1748	336556	343911	2.18	276.98	339617.8	0.91	19.7	339710.8	0.94	19.05
31	U2319	234256	236744	1.06	410.97	235236	0.42	17.02	235975	0.73	18.94
32	Pcb3038	137694	141242	2.57	554.28	139706.2	1.46	27.64	139635.2	1.41	29.05
33	Fnl4461	182566	187409	2.65	830.9	185535.4	1.63	30.43	185509.4	1.61	29.67
34	Rl5934	556045	575437	3.48	1043.95	566166.8	1.82	50.76	566053	1.80	52.5
35	Pla7397	23260728	24166453	3.89	1245.22	2.38E+07	2.48	100.69	2.38E+07	2.24	89.61
36	Usa13509	19982859	20811106	4.14	2016.05	2.04E+07	2.06	365.12	2.04E+07	1.89	326.76
37	Brd14051	469385	486197	3.58	2080.5	478609.6	1.97	375.28	478010	1.84	369.86
38	D18512	645238	669445	3.75	2593.97	658149.2	2.00	654.85	657457.2	1.89	629.14
39	Pla33810	66048945	69533166	5.27	4199.88	68075607	3.07	1959.68	68029226.4	3.00	1998.19
40	Pla85900	142382641	156083025	9.63	8855.13	146495515.6	2.89	7596.18	145526542.6	2.21	7586.6
Average				1.87	650.31		0.81	283.52		0.75	282.49
